# Supplementary figures and images for: Grazing and light modify Silene latifolia responses to nutrients and future climate
Source: PLoS One. 2022 Nov 8;17(11):e0276789. doi: 10.1371/journal.pone.0276789 (PMC9642889; doi:10.1371/journal.pone.0276789)

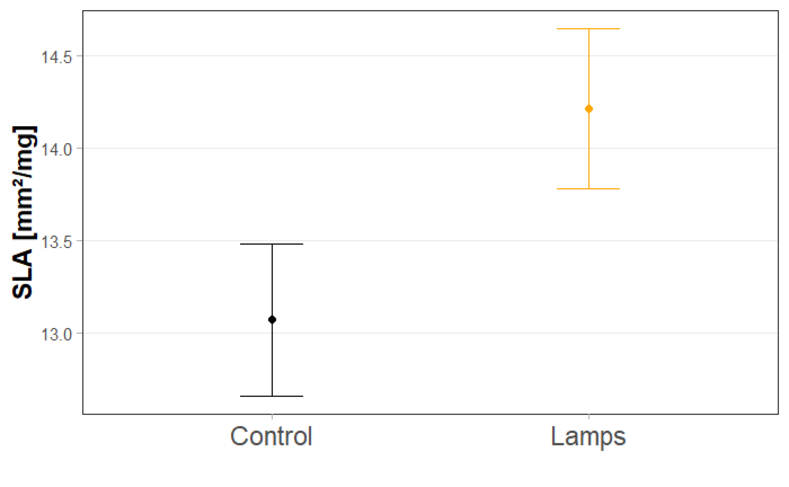

Supplement: S1 Fig — SLA under control and lighted conditions. The data are means ± SE from the leaves collected from inside the fences. (TIF) [file pone.0276789.s001.tif]

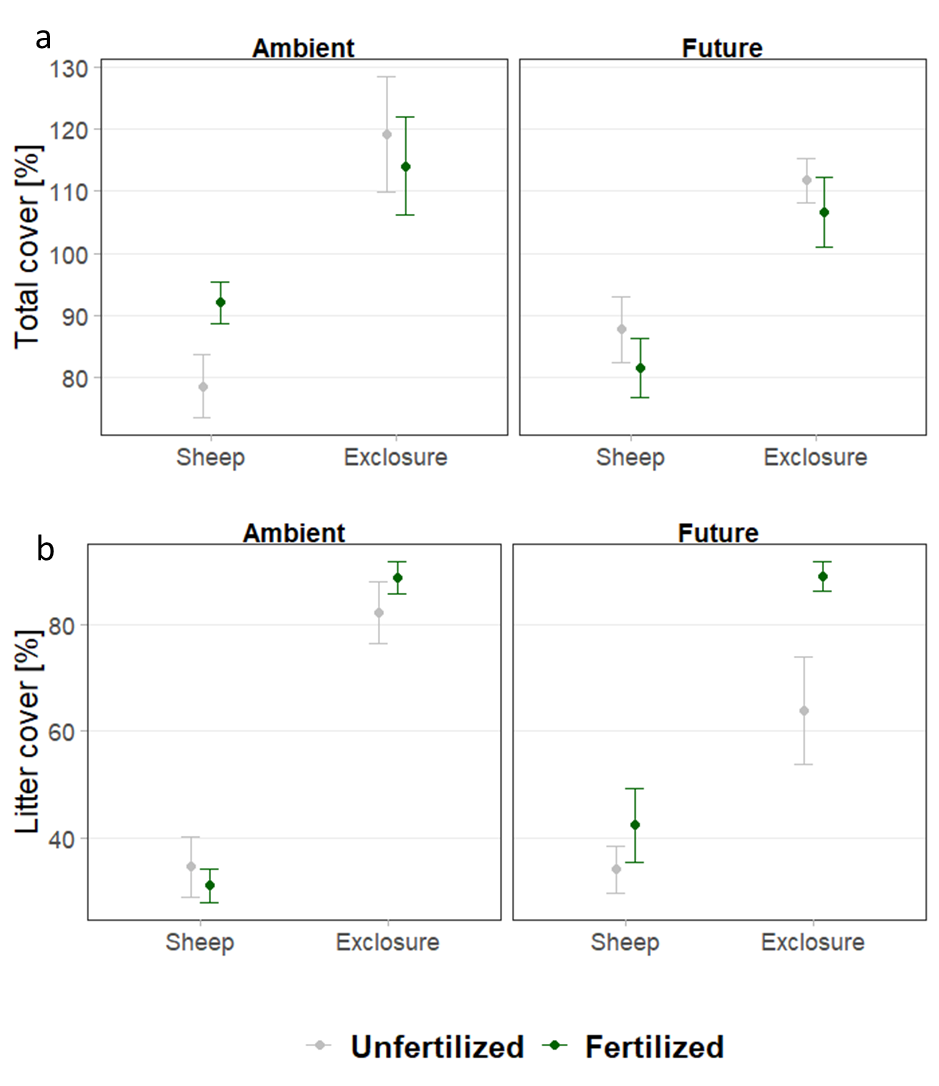

Supplement: S2 Fig — (a) Percent total vegetation cover and (b) percent litter cover in grazed (sheep) and ungrazed (exclosure), unfertilized and fertilized plots under ambient and future climate conditions. Data are means ± SE. Total vegetation cover and litter cover were assessed by visual estimation on the experimental plots in June 2020. (TIF) [file pone.0276789.s002.tif]

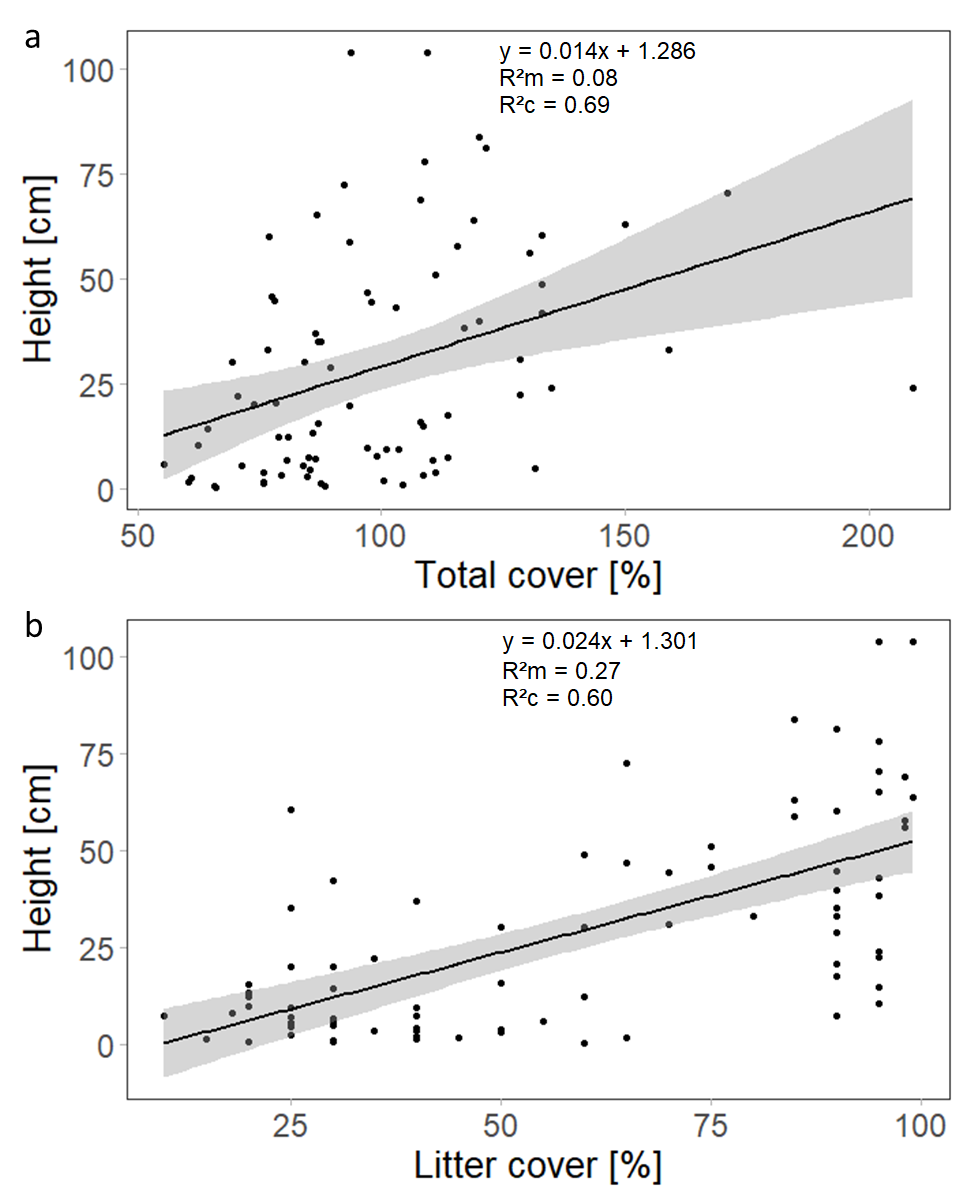

Supplement: S3 Fig — Regression of the visually estimated (a) total vegetation cover and Silene height and (b) litter cover and Silene height. The line represents a regression line with a 95% CI. The regressions are significant (a) F1,74 = 7.58, P = 0.007 (b) F1,56 = 21.74, P < 0.001. Note that the regression equations are based on log-transformed height values, while the y-axis shows not transformed values. Rm (marginal) refers to the amount of variance explained by the fixed effects only, Rc (conditional) refers to the amount of variance explained including the random effects (i.e. plot nested in block). (TIF) [file pone.0276789.s003.tif]

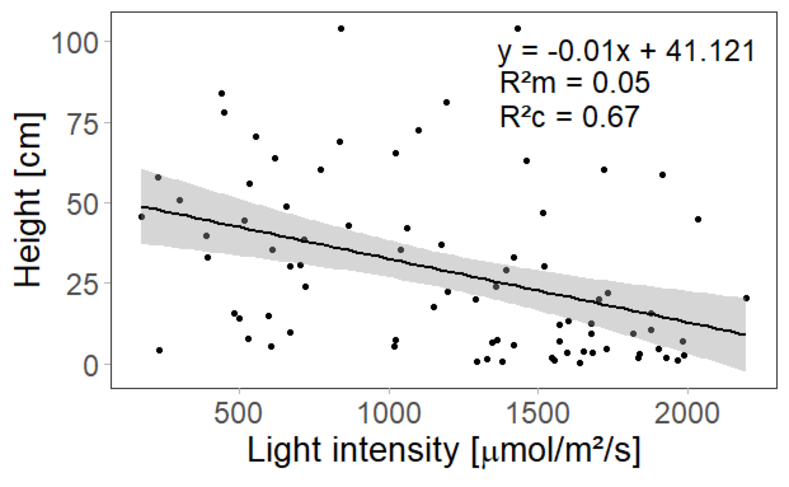

Supplement: S4 Fig — Regression of the light intensity measured approximately 7–10 cm under the lamps and 15–20 cm above ground level and Silene height. The line represents a regression line with a 95% CI. The regression is significant F1,67 = 5.30, P = 0.024. Note that the regression equation is based on log-transformed height values, while the y-axis shows not transformed values. Rm (marginal) refers to the amount of variance explained by the fixed effects only, Rc (conditional) refers to the amount of variance explained including the random effects (i.e. plot nested in block). (TIF) [file pone.0276789.s004.tif]

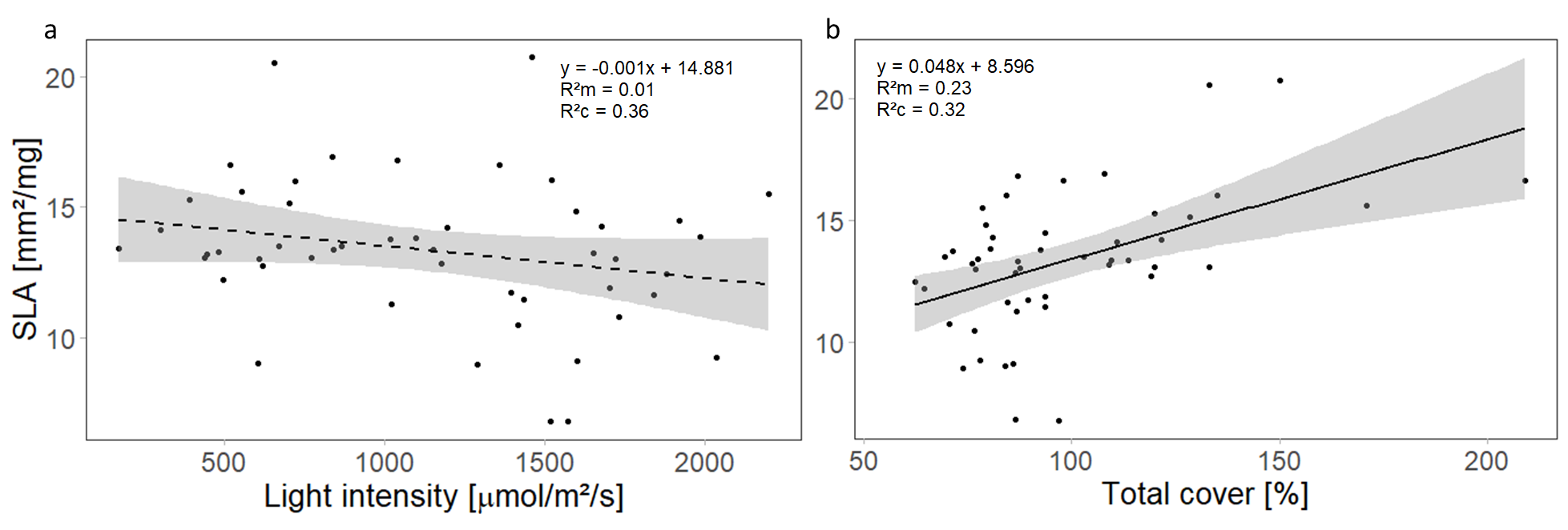

Supplement: S5 Fig — Regression of (a) the light intensity measured approximately 7–10 cm under the lamps and 15–20 cm above ground level and Silene SLA and (b) the visually estimated percentage litter cover and Silene SLA. The lines represent regression lines with a 95% CI. The dashed regression line is not significant (F1,29 = 0.38, P = 0.544), the solid regression line is significant (F1,44 = 13.67, P < 0.001). Rm (marginal) refers to the amount of variance explained by the fixed effects only, Rc (conditional) refers to the amount of variance explained including the random effects (i.e. plot nested in block). (TIF) [file pone.0276789.s005.tif]
