# Supplementary material for: Grazing and light modify Silene latifolia responses to nutrients and future climate
Source: PLoS One. 2022 Nov 8;17(11):e0276789. doi: 10.1371/journal.pone.0276789 (PMC9642889; doi:10.1371/journal.pone.0276789)
Supplement: Attachment — Submitted filename: R1_PLOS_Response letter.docx [file pone.0276789.s008.docx]

**Response letter to PONE-D-22-06013R1**

Reviewer #3: The authors have conducted an effective factorial design to assess a herb species’ response to select environmental changes using transplanted individuals in a test setting. The results are interesting, demonstrating some expected and some unexpected results. I have no overarching concerns

We thank the Reviewer for appreciating our study and for a thoughtful and thorough review. We have done our best to improve the manuscript based on the Reviewer’s comments. Please see our detailed responses below.

(1) The end of the Introduction begins to encroach on Methods (perhaps lines 104 to 113), it seems, citing the ways in which extra light is delivered, traits measured, and data collected. This may be revisited to reduce duplication with Methods.

Our response: Previous reviewers suggested adding this information to better understand the questions and predictions. As our experimental setup is rather complex, we agree with these reviewer suggestions and would like to keep the brief overview of the experimental treatments. Furthermore, we think it is important for the reader to understand our definition of transplant performance, before reading the questions. We have therefore decided to not change this part of the manuscript (please see lines 108 - 112) but are open for editorial advice in this matter.

(2) Line 116. Remove the comma.

Our response: Done.

(3) Lines 116-119. It is odd that hypothesis 1a and an “unless” in it, so to speak. It a little like “’A’ will be true, unless it isn’t.” Not important, but a little unusual. Hypothesis 1b is weak as well, citing both sides of a response, so to speak. I’d suggest editing these to be declarative, citing a specific expected response. Hypothesis 2a is an example of such a statement.

Our response: Thank you for this comment. We fully agree with the reviewer with regard to the prediction 1a and have now specified the expected response (please see lines 116-118). Prediction 1b specification is not straightforward because our climate treatment can lead to different outcomes depending on which season is the most influential for plant growth and survival (these scenarios are explained earlier in the Introduction, please see lines 85-91). We think it is therefore reasonable to integrate both possibilities in the prediction as we have done in prediction 2b.

(4) Line 130. Add an “E” to the longitude

Our response: Thank you for noting this: done.

(5) Line 350. “absence of grazers

Our response: We have added “absence of grazers”.

(6) Regression equations and percent variation explained would be helpful for the later supplemental figures.

Our response: Thank you for this suggestion. We have added the information to the figures in the Supporting Information.
