# Supplementary material for: Grazing and light modify Silene latifolia responses to nutrients and future climate
Source: PLoS One. 2022 Nov 8;17(11):e0276789. doi: 10.1371/journal.pone.0276789 (PMC9642889; doi:10.1371/journal.pone.0276789)
Supplement: Attachment — Submitted filename: Reviewer Comments Directed to Author.docx [file pone.0276789.s006.docx]

This paper study plant responses to interactions among climate, nutrients, grazing and light. This research is helpful for evaluating ecosystem species competition, community composition and biodiversity.

The manuscript for this paper is technically sound, and the data support the conclusions. Appropriate and rigorous statistical analysis was performed in the manuscript of this paper. For example, the manuscript used a linear mixed-effects model for data analysis, nesting plots within blocks, and taking into account autocorrelations. All data available in the manuscript. The manuscript is presented in an intelligible manner and written in standard English, with minor grammatical errors.

However, this article has some deficiencies as follows:

1. In the abstract part, the author describes the important content of the paper very clearly, but the description is too cumbersome. It is recommended to refine the abstract part. For example, there is no need to emphasize “an abundant and widespread grassland forb in Europe” in the abstract.
2. Statistical analysis needs to be described in as much detail as possible so that other scholars can repeat it.
3. It is suggested that the description of the results section needs to be improved, and should be a perfect elaboration of the four scientific questions raised above.

In summary, my suggestion is to make a minor revision of this manuscript this manuscript cannot be accepted.
